# Spatial evaluation of healthcare accessibility across archipelagic communities of Maluku Province, Indonesia

**DOI:** 10.1371/journal.pgph.0001600

**Published:** 2023-03-09

**Authors:** Yanti Leosari, Johnny Albert Uelmen, Ryan Marc Carney

**Affiliations:** 1 College of Public Health, University of South Florida, Tampa, Florida, United States of America; 2 Department of Integrative Biology, University of South Florida, Tampa, Florida, United States of America; University College London, UNITED KINGDOM

## Abstract

The Maluku Province is an underdeveloped region in Indonesia with over 1,340 scattered islands. Due to the limited health facilities and transportation infrastructure, access to healthcare is very challenging. Here, we combined data from various sources to locate the population clusters, health facilities, roads, and ports/docks, and then utilize geographic information systems (GIS) to estimate distances from residents to health facilities. Health workforce distribution data was then integrated to elucidate overall healthcare equity among districts in the province. The average distances to puskesmas (primary health clinics) were 8.89 km (by land) and 18.43 km (by land and water) respectively, and the average distances to hospitals were 56.19 km (by land) and 73.09 km (by land and water), with large disparities within and among districts. Analysis of health workforce data shows that 65% of 207 puskesmas lack physicians, while 49% lack midwives. Ambon, Tual, and Southeast Maluku have the highest health equity, while East Ceram, Buru, and South Buru have the lowest. In general, this study demonstrates the utility of GIS and spatial analyses, which can help identify problem areas in healthcare accessibility and equity in archipelago settings, and provide recommendations to stakeholders such as public health officials and district administrators.

## Introduction

Since 2005, the World Health Organization (WHO) has called for a resolution to provide universal health coverage to all people and communities [1]. However, delivering effective and quality healthcare services remains a common and persistent problem within the global healthcare ecosystem [2]. Access to healthcare revolves around bringing people into contact with the necessary facilities, expertise, and treatment at times of need. Long distance and time needed to access health facilities is a central issue that contributes to the slow and uneven progress towards appropriate promotive, preventive, curative, and rehabilitative care at affordable cost [3, 4]. Longer distances also detract from an individual’s willingness to seek medical care, likely resulting in poorer health outcomes.

Low-and-middle-income countries with large geographic territories are usually characterized by widely dispersed populations and scant medical resources [3]. The nearest health facility is often located at great distances from the population. Coupled with poor road networks and inadequate transportation infrastructure, this often leads to late arrivals, delayed care or missed opportunities, and eventually worse health outcomes [5].

Indonesia is the world’s largest archipelago and fourth most populous nation, which consists of ≥17,000 islands (of which 6,000 are inhabited) [6]. The total population of 270 million is unevenly distributed: more than half reside on Java island, which constitutes only 7% of the country’s total land mass, while Papua island represents 22% of the total land mass but only 3% of the population [7, 8]. Therefore, development efforts are highly centralized in Java, leaving the outer islands–especially in the eastern part of the country–relatively neglected.

Besides distance, health workforce is another major factor that determines accessibility to health services. Even if a person reaches a healthcare center, availability of care is impaired by shortage of trained health workers [3]. There is fewer than one doctor for every 2,000 people in Southeast Asia and Sub-Saharan Africa, while the ratio is six times higher in developed countries [9]. Indonesia operates a three-tiered health system, which relies heavily on primary healthcare at the grassroots level. Public primary health centers (puskesmas) serve as the main gateway to the healthcare system by providing basic health services to the population [10], particularly in remote and rural areas where access to hospitals is very limited. A national regulation stipulates that every outpatient puskesmas have at least one physician and those providing inpatient services have at least two [11].

Despite the various efforts to mobilize a more equitable distribution of health workforce in the past decades, Indonesia’s archipelagic geography and unequal development have discouraged many health workers from serving in rural and remote regions [12]. Rokx et al. estimated that only 20% of physicians were based in rural areas, where nearly 70% of the population resided [13]. The ratio of physician to population was only 6 per 100,000 in rural areas, while in urban areas the ratio was 36 per 100,000. In Java-Bali, the most developed and populous region in the country, 85.2% of puskesmas have a sufficient and even excessive number of physicians. This situation is in stark contrast to the Nusa Tenggara-Maluku-Papua region, where 52.41% of puskesmas do not have enough physicians [14].

The province of Maluku (Moluccas) is one of the least developed regions in Indonesia. In 2015, its per capita GDP was the second lowest out of 33 provinces. In terms of educational attainment, only 31% of the residents completed high school and 7.3% have a university degree [15]. The administration is divided into 11 districts, three of which are categorized as disadvantaged and outermost islands regions [16]. Maluku’s 712,479 km^2^ territory is mostly made up of water (92.4%), and the land area (7.6%) is scattered into 1,340 islands where 1,848,923 people reside [16, 17]. The topography varies from coastal, low and swampy, hilly, to mountainous regions covered by dense evergreen forests [18]. Many of the island clusters are isolated from one another, making connectivity among them very challenging.

This highly dispersed nature of the Moluccan archipelago, along with the underdeveloped transportation system in the region have caused serious disruption to healthcare delivery. Puskesmas are available in all 11 districts of the province, however, many still lack basic infrastructure, medical equipment, and human resources. Basic diagnostic tests such as hemoglobin, blood glucose, and urine tests, as well as essential medicines and vaccines, are not available in more than half of the puskesmas [19]. A report from the WHO revealed that 25.4% of subdistricts in Maluku are without puskesmas, and more than one third of puskesmas do not satisfy the criteria for basic amenities readiness [20].

While the actual number is unknown, private primary care facilities (clinics and private practices) are generally rare and less utilized in eastern Indonesia [21]. Hospitals, as the secondary health facilities, are usually located in the district capitals, while more advanced hospitals tend to be concentrated in a few larger cities. This situation makes healthcare access very challenging for the poor and disadvantaged communities living in isolated islands when they need more complicated medical procedures [22]. Consequently, seven of Maluku’s eleven districts are included in the list of worst-performing regions in public health [16]. The province has the lowest coverage of facility-based childbirth (45% compared to the national target of 82%). Similarly, the coverage of antenatal care and post-natal care for newborns was reported to be less than 50%, and fewer than one in three children receive complete basic vaccination [20].

Healthcare research and planning, especially in the area of measuring access to and coverage of health services, has increasingly relied on geographic information systems (GIS) [2]. GIS outweighs conventional methods (table, graph, diagram) due to its powerful spatial visualization and analyses, thus allowing for quick assessments of trends and interrelationships [23]. There have been numerous studies that employed a GIS approach to investigate accessibility to health services in developing countries. For example, Mansour (2016) examined the spatial pattern of public health facilities distribution to locate underserved regions in Saudi Arabia [24]. Another study associated straight-line distances between population clusters and health facilities with provider-to-population ratios, to present disparity in healthcare accessibility among subdistricts in Bhutan [25]. Luqman and Khan (2021) utilized network analysis to determine travel time to health facilities and identify disadvantaged populations in Egypt [26]. Other researchers applied a similar method to estimate primary health care coverage among the Mozambican populations [27]. More commonly used are floating catchment area (FCA) methods to estimate healthcare accessibility, involving travel distance within well-established administrative districts [28, 29].

However, most of these studies were conducted in a mainland setting, leaving a gap in our understanding with respect to archipelagic settings, particularly where transportation networks are inadequate to support quick mobility. This study aims to fill these gaps by estimating distances from residents to their adjacent health facilities over both land and water, and by integrating health workforce distribution data to assess the overall healthcare equity. Our findings on healthcare accessibility among the understudied and vulnerable population of Maluku Province are expected to help facilitate better planning and a more equitable distribution of resources.

## Methods

### Study area

Geographic coordinates of puskesmas and hospitals in Maluku were obtained from the Ministry of Health [30], then exported into ArcMap 10.8.1 (Environmental Health Research Institute, Inc. Redlands, CA, USA) and converted (WGS 1984 to Indonesian 1974 UTM Zone 52S) to create a distribution map of health facilities. The province has a total of 207 puskesmas and 28 general hospitals spreading across 11 districts [30]. Population data based on the 2020 census was acquired from the Central Bureau of Statistics [31]. For every district, we divided the total area of each residence polygon by the total area of all residence polygons of the district, then multiplied the ratio by the district’s total population. This gave us the estimated population residing in each residential cluster in every district.

### Public health metrics

Real-time data of the number of healthcare workers in each hospital and puskesmas across the country was taken from The Agency for Health Human Resource Planning and Development of the Ministry of Health [32, 33]. Physicians, nurses, and midwives were selected as essential healthcare workers due to their critical roles in providing basic essential healthcare services. We then used the national recommended standard [34] to compare the ratio of health workforce to the population in Maluku Province.

### Travel routes from residences to healthcare facilities by land

Shapefiles containing high-density urban areas were provided by the Geospatial Information Agency of Indonesia [35]. However, these urban areas did not capture several small towns and residences located in rural regions of the province. To account for these locations, we used 2021 World Basemap satellite imagery from ArcMap (Esri, DigitalGlobe, GeoEye, i-cubed, USDA FSA, USGS, AEX, Getmapping, Aerogrid, IGN, IGP, swisstopo, and the GIS User Community) imagery to manually locate and trace households for each district in Maluku Province. This manual tracing approach added an additional 298 residential locations to the existing 2,029 provided from the government. The centroid of all residential shapefiles was calculated and converted to a point for future connectivity analyses.

We integrated the Maluku Province road network, also provided by the Geospatial Information Agency [35], as the main path that individuals take to travel from their residences to the nearest healthcare facility. Routes were determined by using the Closest Facility tool in the Network Analyst extension. Per Indonesian regulation [36], residents utilizing services from public healthcare facilities are required to visit locations in the district they reside in. Likewise, puskesmas are also required to refer patients to hospitals within the same district as the first option. All travel routes estimated in our analyses followed this protocol.

### Travel routes from residences to healthcare facilities by water

Residential areas that do not have accessibility to a healthcare facility via existing road network were determined under the following conditions: 1) residences located >5 km from the nearest road, or 2) whose island of residence did not contain a hospital or puskesmas. In these residential areas, individuals are required to travel to the nearest accessible water access point. Extensive review found no previous spatial documentation of docks and/or ports in Maluku district to use for our analysis. In addition, nearly all remote residences situated along or near beaches used small watercraft for transportation, without existing infrastructure to support docks or ports (small boats land and depart directly from sandy beach). To remedy this, we performed the same manual approach conducted above and identified 439 ports and docks, as well as 736 beaches that were either directly adjacent to or within 500 meters of a residential area. From this newly created point shapefile, we were able to approximate the nearest water access point, either by dock, port, or by beach, where individuals could then reach the nearest puskesmas or hospital by boat.

**Fig 1:**
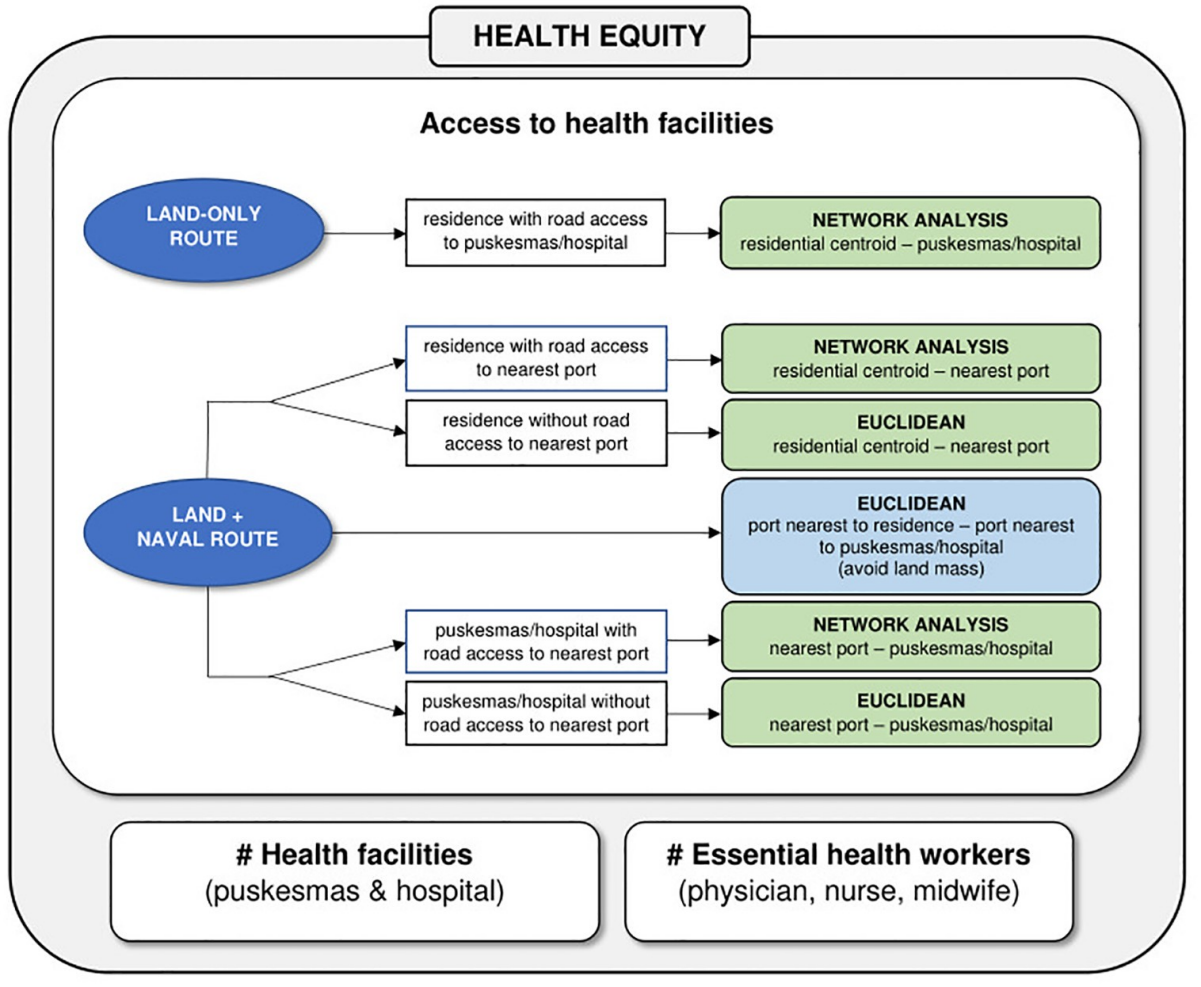
Diagram illustrating the three components of health equity considered in the present study, including the processing steps for calculating spatial accessibility to health facilities. Green boxes denote land route, blue box denotes naval route.

Nearly all puskesmas and hospitals were located next to ports or docks. Using the Near tool in ArcMap, we created an additional point shapefile indicating the nearest port or dock to a puskesmas or hospital for all districts in Maluku Province. For any given hospital or puskesmas, the closest port or dock would serve as the main hub for individuals requiring naval transportation from distant islands and/or remote locations. Naval routes were estimated by finding the Euclidean distance from distant or remote port, dock, or beach to the closest dock or port to a respective hospital or puskesmas. However, the Euclidean distance was modified to avoid all land barriers and represent travel solely via water (Fig 1).

### Statistical analysis

**Fig 2:**
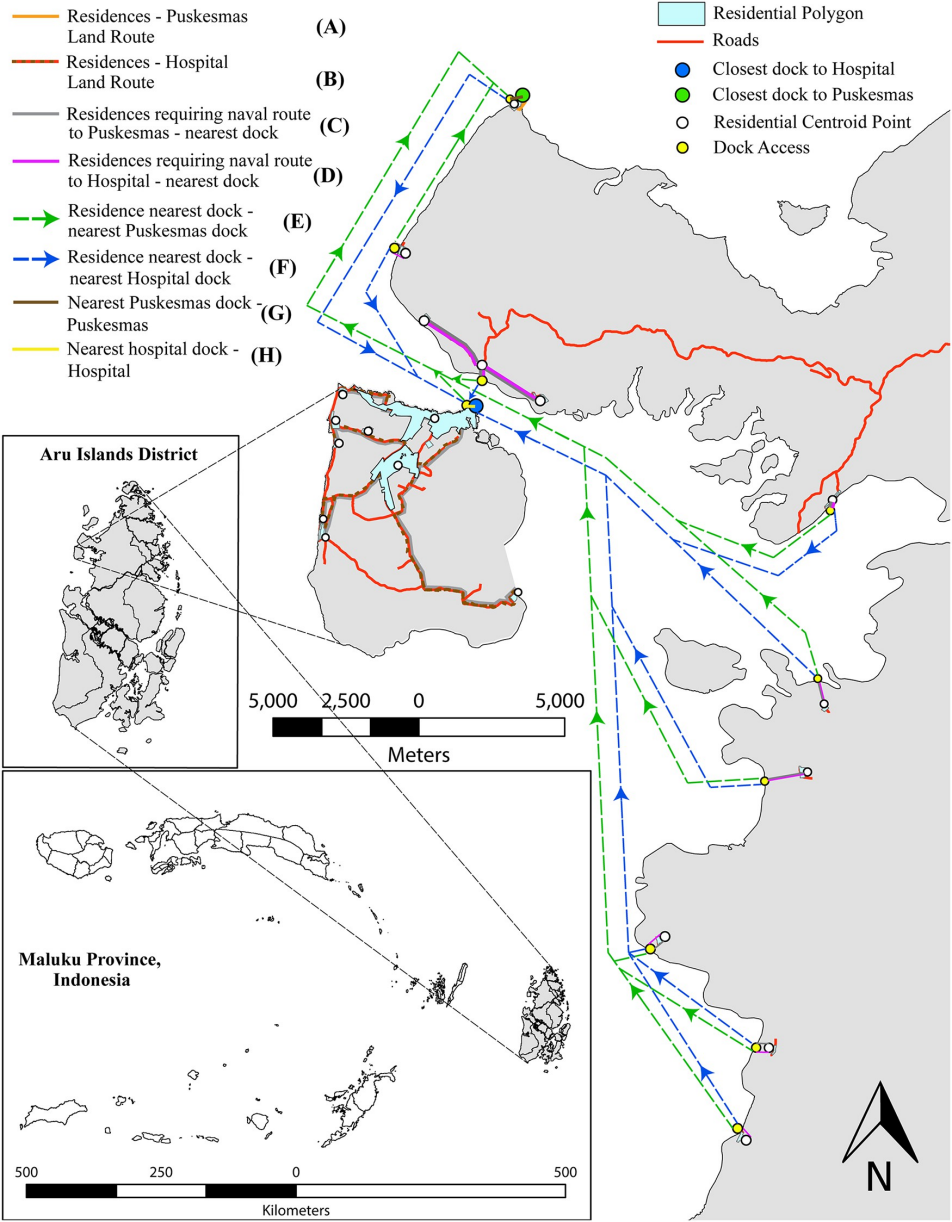
Example of route types and their corresponding values (in reference to Table 2). Base map provided by GADM https://gadm.org/maps/IDN.html. License https://gadm.org/license.html.

To estimate the most practical route individuals would need to travel from their residences to a healthcare facility, we calculated the shortest distance following the established road network using the Network Analysis tool in ArcMap (labeled as route A, Fig 2). For residential areas located on islands with adequate access to existing road networks connecting to a hospital or puskesmas, the land distance calculated from our network analysis was averaged by district. However, due to the heterogeneity of land and water features located throughout the Maluku archipelago, routes were frequently disconnected or broken by naturally occurring features in topography (high elevation, thick jungle, various waterways, etc.). For these residential locations, we analyzed each of the following routes separately by district: 1) residential centroid to nearest port (route B), 2) nearest port to the port closest to a nearest hospital (route C) or puskesmas (route D), 3) distance from nearest hospital or puskesmas port to respective healthcare facility (not shown in Fig 2 due to the very small distance on map). An example of these separate routes and how they were integrated into an average is presented in Fig 2. Average travel route distances were compiled by district as residence to hospital and puskesmas for both land (only) and land with naval transportation (Table 1).

**Table 1:** Overview of key public health characteristics by district. These values provide the foundation for comparison of overall healthcare equity for individuals within each district.

Key Public Health Characteristics	Ambon	Aru Islands	Buru	Central Maluku	East Ceram	South Buru	Southeast Maluku	Southwest Maluku	Tual	West Ceram	West Southeast Maluku	MALUKU
Healthcare Centers												
Puskesmas	22	27	10	33	19	12	18	21	15	17	13	207
Hospitals	9	1	1	4	1	1	3	1	1	1	3	26
Healthcare Providers												
Physicians	125	12	14	69	24	5	31	14	22	16	38	370
Nurses	899	257	298	592	404	179	336	242	249	349	243	4,048
Midwives	253	59	112	407	149	83	115	80	105	193	44	1,600
Route to Healthcare Center Traveled by Population												
Population	347,288	102,237	135,238	425,631	137,972	75,410	121,511	81,928	88,280	209,856	123,572	1,848,923
Individuals who can reach puskesmas by land	347,288 (100%)	63,342 (61.96%)	115,982 (85.76%)	411,834 (96.76%)	125,378 (90.87%)	71,800 (95.21%)	112,533 (92.61%)	39,934 (48.74%)	84,242 (95.43%)	179,359 (85.47%)	107,273 (86.81%)	1,658,965 (89.73%)
Individuals who can reach hospital by land	347,288 (100%)	37,212 (36.40%)	115,982 (85.76%)	386,641 (90.84%)	94,118 (68.22%)	55,085 (73.05%)	102,988 (84.76%)	18,505 (22.59%)	75,011 (84.97%)	173,637 (82.74%)	89,060 (72.07%)	1,495,527 (80.89%)
Individuals who require land and naval routes to reach puskesmas	-	38,895 (38.04%)	19,256 (14.24%)	13,797 (3.24%)	12,594 (9.13%)	3,610 (4.79%)	8,978 (7.39%)	41,994 (51.26%)	4,038 (4.57%)	30,497 (14.53%)	16,299 (13.19%0	189,958 (10.27%)
Individuals who require land and naval routes to reach hospital	-	65,025 (63.60%)	19,256 (14.24%)	38,990 (9.16%)	43,854 (31.78%)	20,325 (26.95%)	18,523 (15.24%)	63,423 (77.41%)	13,269 (15.03%)	36,219 (17.26%)	34,512 (27.93%)	353,396 (19.11%)
Mean distance traveled by route type (km)												
Puskesmas by land	2.98	2.88	22.3	11.8	7.69	12.13	5.61	5.89	1.6	12.11	12.75	8.89
Hospital by land	5.96	4.87	63.39	85.71	254.18	92.43	22.37	2.53	7.76	44.31	34.56	56.19
Puskesmas by land and naval routes	-	16.31	26.04	32.18	15.36	19.19	12.12	19.39	11.02	15.92	16.76	18.43
Hospital by land and naval routes	-	99.10	56.20	73.83	107.33	60.13	25.46	178.58	64.78	25.94	39.50	73.09
All routes	4.47	30.79	41.98	50.88	96.14	45.97	16.39	51.60	21.29	24.57	25.89	37.27

In this example, a small subsection of Aru Islands district is provided to demonstrate the routes most likely taken by residents (indicated by yellow centroid points) to reach either their nearest hospital (blue dot) or puskesmas (green dot). Note road networks (indicated by red lines) sparsity and lack of connection to nearby residential areas, requiring the frequent use of docks and naval routes to reach healthcare facilities.

Healthcare resources by district was estimated by two factors: 1) the total number of puskesmas and hospitals and 2) the total number of essential healthcare workers employed at those facilities. Due to the differences in healthcare capabilities and services that are rendered by medical provider type, healthcare coverage is weighted based on the Indonesian national standard (physicians = 1:2500 residents; midwives = 1:1000 residents; nurses = 1:855 residents translating to a weighted factor of 2.92, 1.17, and 1, respectively). Healthcare resources in each district was calculated as

$$\frac{Population}{\Sigma \left(\mu a,b\right)},$$

where:

μ = average(# physicians*2.92)+(# midwives*1.17)+(# nurses*1),

a = # puskesmas within a district,

b = # hospital within a district.

Total distance to reach health facilities (puskesmas and hospital) through land and naval route by district was calculated as

$$\Sigma \left(cd,ef\right),$$

where:

c = % individuals who can reach puskesmas by each type of route

d = mean distance traveled to puskesmas by each type of route

e = % individuals who can reach hospital by each type of route

f = mean distance traveled to hospital by each type of route

Overall healthcare equity by district was estimated as the mean availability of health workers (by puskesmas and hospitals for each district) as a function of total distance a resident would need to travel. This is expressed as

$$\frac{z}{\mu \left(x,y\right)},$$

where:

x and y = average puskesmas and hospital healthcare availability (weighted by number and type of healthcare workers employed), and z = the total distance traveled by population for all route types.

Healthcare equity is inversely related to the travel estimate value (low is excellent, high is severe).

Mean values for distance traveled, population to healthcare workers, and healthcare visits were compared by district using Student’s t-test (p > 0.05) in JMP Version 16.0 (SAS Institute Inc., Cary, NC, 1989–2022). Unique letters among each group indicate mean values that are statistically different from the other group’s mean values and are arranged in descending order (A = highest mean value, B is next highest mean value, and so on). Traditional floating catchment area (FCA) methods were not used in this analysis due to the lack of information and data availability regarding uniform travel distances, the disconnect from islands and the use of naval routes in conjunction with land routes, as well as the sparsity of healthcare facilities. However, we conducted a two-step floating catchment area (2SFCA) analysis as a comparison to our results. The methodology and results are located in the Supplemental Materials.

## Results

**Fig 3:**
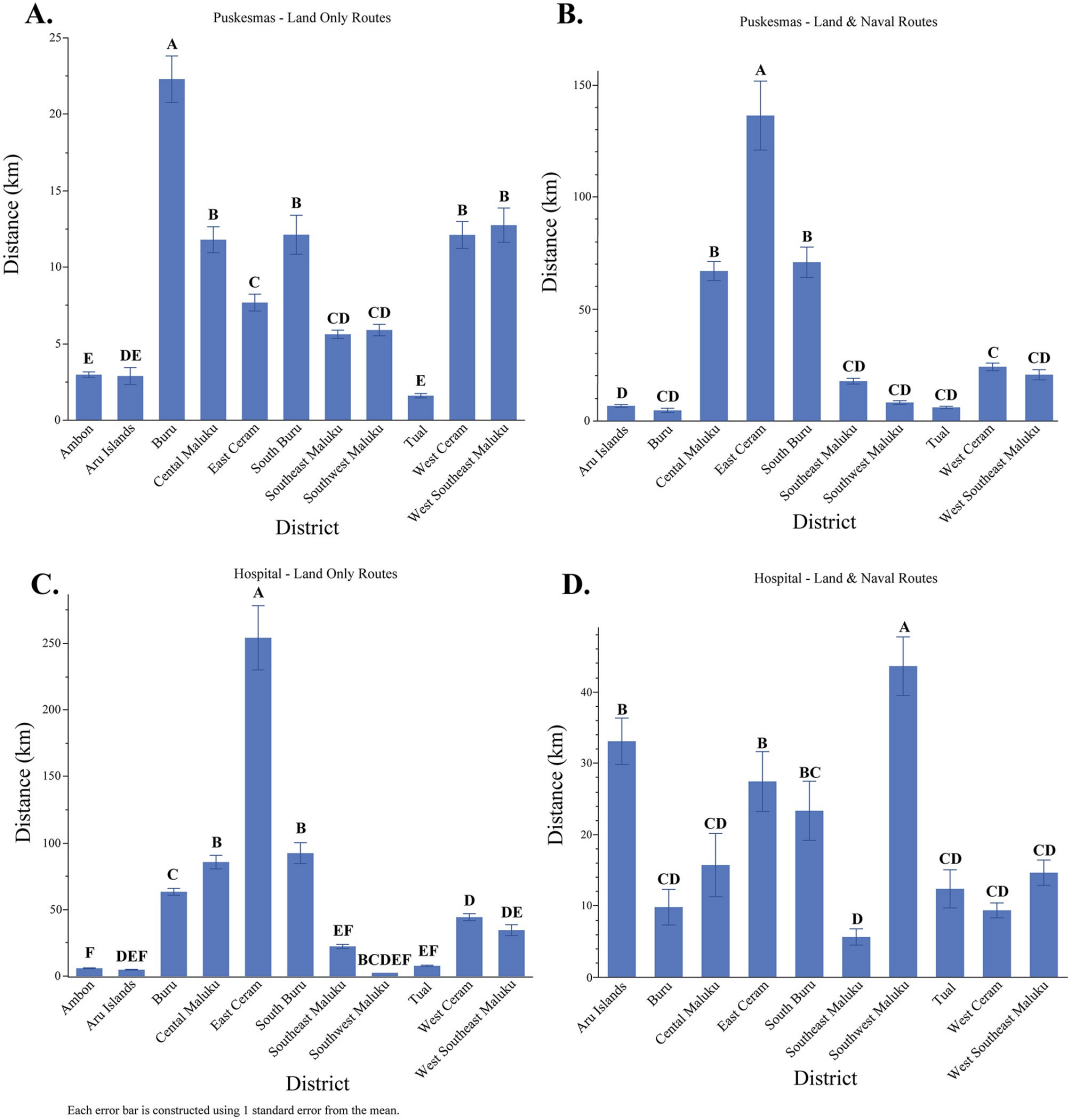
Total mean distance length (km) by healthcare facility (hospital and puskesmas) and route type (land and naval routes). Within-group statistical comparisons were conducted by using Student’s t-test; unique letters indicate significantly different mean values (*p* < 0.05).

The majority of the population in Maluku has access to puskesmas by land (89.73%) with an average distance of 8.89 km, while the remaining 10.23% has to travel an average distance of 18.43 km over combined land and naval routes (Table 1). However, large disparities exist due to the great variation in total area, population density, and health facilities distribution in each district (Fig 3). In Buru, land travel to puskesmas is ~22 km and accessible by 86% of the population, while in Aru Islands, the distance is much shorter (~3 km) but accessible by only 62% of the residents. The whole population of Ambon can reach the nearest puskesmas by traveling ~3 km solely on land, but more than half of Southwest Maluku residents must travel an average of 19 km on land and water.

Access to hospitals is more challenging for patients. Although 81% of the population has access to road infrastructure to visit a hospital, the average distance traveled is 56.19 km. The remaining 19% must travel 73 km through land and naval routes. Inequality is also apparent in the fact that most districts only have one hospital to serve the entire population (Fig 3). In Ambon, the nearest hospital is located within 6 km of a given resident, whereas two-thirds of the population in East Ceram who have land access to a hospital need to travel 254 km, and only about a quarter of people in Southwest Maluku have access to a hospital by land.

Central Maluku has the longest naval routes to puskesmas and hospitals, at 177.60 km and 290.48 km respectively (Table 2). On average, residents who has no direct land access to puskesmas in Central Maluku must travel 22.95 km, whereas those in Tual only need to travel 8.36 km. The shortest average naval route to a hospital is found in West Ceram (23.35 km), while the longest is found in Southwest Maluku (174.93 km).

**Table 2:** Descriptive statistics summarizing route characteristics by type (A-H, Fig 2) and district.

District (# residential centroid)	Summary statistic	Route Type* (km)							
		Land route		Land and naval route					
		Residences–puskesmas	Residences–hospital	Residences requiring naval route to puskesmas–nearest dock	Residences requiring naval route to hospital–nearest dock	Residence nearest dock–nearest puskesmas dock	Residence nearest dock–nearest hospital dock	Nearest puskesmas dock–puskesmas	Nearest hospital dock–hospital
		A	B	C	D	E	F	G	H
Ambon (149)	N	149	149						
	Mean	2.98	5.96						
	Standard Error	0.18	0.36						
	Median	2.42	4.9						
	Minimum	0.07	0.28						
	Maximum	8.84	16.33						
Aru Islands (153)	N	52	13	101	140	84	88	23	1
	Mean	2.88	4.87	2.00	1.73	12.17	91.39	2.14	5.98
	Standard Error	0.56	0.35	0.45	0.34	0.76	5.21	1.06	
	Median	0.62	4.9	0.19	0.19	11.84	90.86	0.36	5.98
	Minimum	0	2.45	0	0	0.66	0.98	0	
	Maximum	14	7.15	27.51	27.51	31.17	251.18	22.3	
Buru (280)	N	220	220	60	60	8	11	9	1
	Mean	22.3	63.39	2.81	2.81	18.19	52.83	5.04	0.56
	Standard Error	1.52	2.58	0.56	0.56	5.12	11.84	2.23	
	Median	16.06	62.76	0.98	0.98	10.08	43.86	1.78	0.56
	Minimum	0.1	0.03	0.07	0.07	5.61	16.7	0.05	
	Maximum	90.53	146.67	21.41	21.41	36.54	116.99	19.86	
Central Maluku (342)	N	286	259	56	83	31	32	34	4
	Mean	11.8	85.71	0.46	0.37	29.55	71.67	2.17	1.79
	Standard Error	0.85	5.05	0.09	0.06	7.42	18.29	0.94	0.57
	Median	6.76	51.56	0.18	0.22	12.63	21.62	80	2.14
	Minimum	0.02	0.45	0	0	1.17	1.74	0.3	0.21
	Maximum	74.45	276.15	3.13	3.27	177.6	290.48	32.19	2.67
East Ceram (244)	N	188	108	56	136	41	53	18	1
	Mean	7.69	254.18	1.29	1.05	10.26	103.67	3.81	2.61
	Standard Error	0.55	24.15	0.35	0.16	0.92	11.15	3.29	
	Median	5.67	103.69	0.33	0.45	9.25	74.08	0.45	2.61
	Minimum	0.01	2.43	0	0	1.87	6.49	0.05	
	Maximum	38.64	632.07	13.24	13.24	23.95	249.22	59.73	
South Buru (120)	N	110	90	10	30	20	25	12	1
	Mean	12.13	92.43	0.30	1.13	8.74	57.18	10.15	1.82
	Standard Error	1.27	7.81	0.21	0.48	0.84	6.66	6.51	
	Median	7.68	73	0.03	0.17	8.98	53.24	0.46	1.82
	Minimum	0.06	0.91	0	0	1.74	3.23	0.17	
	Maximum	57.25	262.89	2.09	9.56	14.74	125.42	58.65	
Southeast Maluku (252)	N	213	172	39	80	20	27	17	3
	Mean	5.61	22.37	0.36	0.60	10.99	24.21	0.77	0.65
	Standard Error	0.27	1.54	0.07	0.10	1.89	3.52	0.25	0.18
	Median	4.76	16.26	0.15	0.17	8.48	15.85	0.35	0.72
	Minimum	0	0.08	0	0	2.48	2.3	0.1	0.31
	Maximum	14.49	79.54	2.13	4.45	34.8	63.4	3.47	0.93
Southwest Maluku (299)	N	216	1	83	298	66	98	17	1
	Mean	5.89	2.53	1.70	2.99	16.39	174.93	1.3	0.66
	Standard Error	0.37		0.41	0.20	1.22	8.4	0.67	
	Median	5.06	2.53	0.24	1.16	15.67	211.31	0.13	0.66
	Minimum	0	2.53	0	0	0.9	4.65	0	
	Maximum	28.12	2.53	15.95	15.95	47.51	282.34	10.06	
Tual (110)	N	90	46	20	64	11	21	15	1
	Mean	1.6	7.76	0.53	0.94	8.36	54.62	2.13	9.22
	Standard Error	0.14	0.53	0.24	0.14	1.32	7.45	0.75	
	Median	1.39	7.81	0.20	0.38	6.95	55.53	0.82	9.22
	Minimum	0	0.51	0	0	1.96	3.02	0.03	
	Maximum	7.28	14.82	4.69	4.69	15.83	95.32	9.15	
West Ceram (231)	N	148	137	83	94	67	67	14	1
	Mean	12.11	44.31	0.38	0.37	12.05	23.35	3.49	2.22
	Standard Error	0.88	2.57	0.08	0.07	0.87	1.6	1.91	
	Median	9.09	39.89	0.14	0.15	11.49	25.34	1.1	2.22
	Minimum	0.07	0.12	0	0	1.75	2.26	0.24	
	Maximum	53.77	134.61	4.27	4.27	28.3	54.18	27.15	
West Southeast Maluku (146)	N	104	75	42	71	41	52	13	3
	Mean	12.75	34.56	0.49	0.49	15.53	38.29	0.74	0.72
	Standard Error	1.12	4.11	0.22	0.14	1.64	2.26	0.32	0.08
	Median	10.55	21.37	0.10	0.14	14.83	35.11	0.28	0.79
	Minimum	0	0.2	0	0	2.29	8.11	0.06	0.55
	Maximum	46.47	116.52	7.69	7.69	59.17	76.65	4.17	0.8

*Refer to Fig 2 for example route type

Analysis of the data on health workers indicates that there is a total of 370 physicians, 4,048 nurses, and 1,600 midwives employed in 207 puskesmas and 28 hospitals in Maluku Province. Of these, 122 physicians (33%), 2,243 nurses (55%), and 1,142 midwives (71%) work in puskesmas. Furthermore, physicians are available in only 86 puskesmas, leaving the remaining 121 puskesmas operated by health workers with lower clinical competency. All districts average less than one physician per puskesmas, except for West Southeast Maluku, where each puskesmas has an average of two physicians. South Buru is the only district that does not have any physician in all of its puskesmas. Of the 248 physicians practicing in hospitals, 112 are specialists while the remaining 136 are general practitioners (GPs).

**Fig 4:**
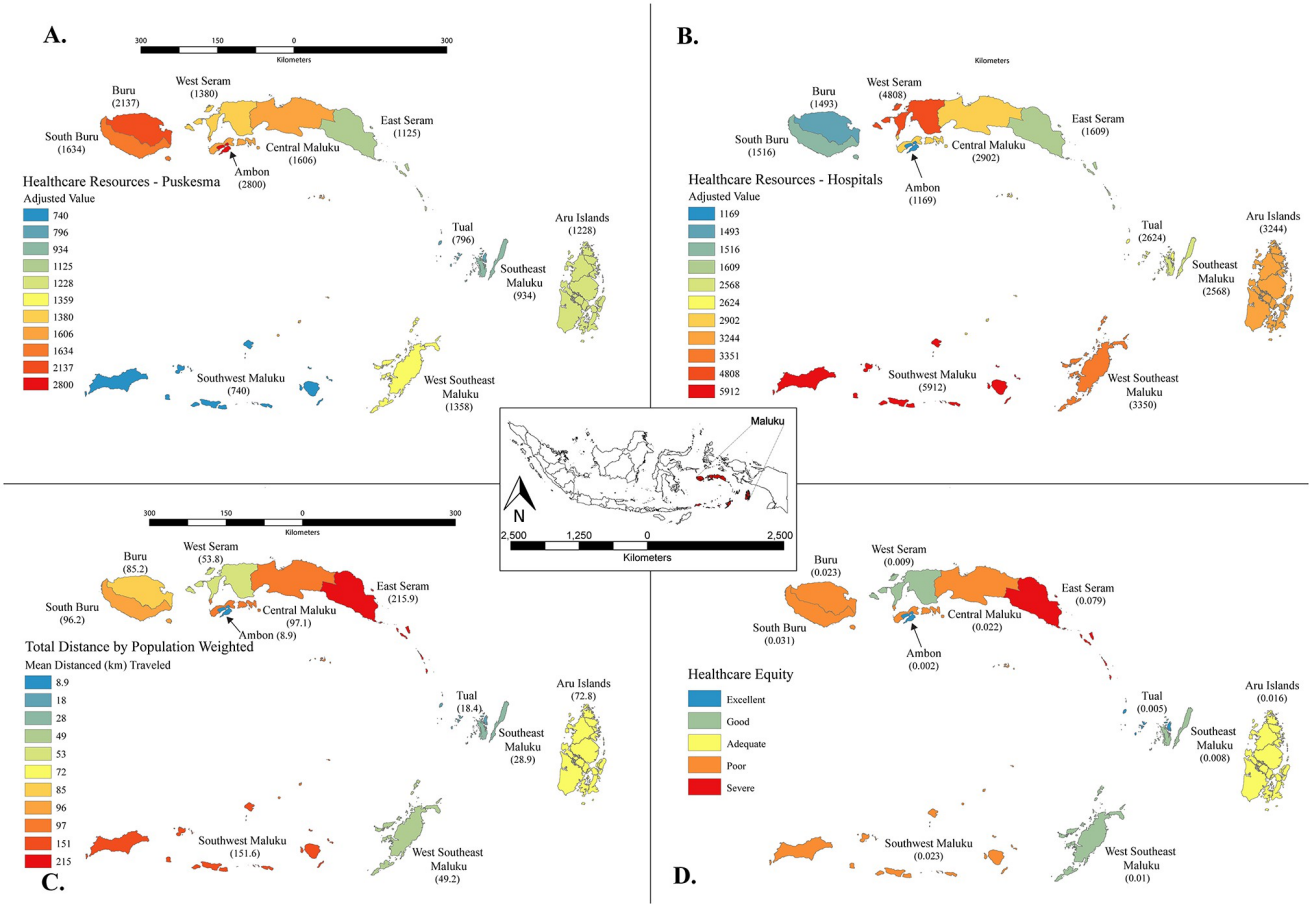
Key healthcare characteristics by district. Health workers in puskesmas (A) and hospitals (B) (weighted by type of health workers and their ratio to population), and total distance traveled to healthcare center (weighted by proportion of population with access by land only, and proportion of population that require access by land and naval routes) (C). Overall healthcare equity by district (D) provides a spectrum of values ranging from excellent (low values) to severe (high values), as a function of healthcare coverage weighted by provider availability and distance required to travel for care across Maluku Province, Indonesia. Base map provided by GADM https://gadm.org/maps/IDN.html. License https://gadm.org/license.html.

Fig 4A and 4B show the distribution of health facilities and essential health workers as a ratio to population across puskesmas and hospitals in Maluku Province. In general, it appears that the value in puskesmas tends to be inversely related to that in hospitals. Ambon and Southwest Maluku are most notable as they occupy the extreme and opposite values in both cases. Similarly, Buru and South Buru rank toward the bottom in puskesmas but near the top in hospitals.

The value of distances weighted by the type of route (land and water) that the population must take in order to access health services is presented in Fig 4C. Smaller districts like Ambon, Tual, and Southeast Maluku have shorter distances than those with larger area and scattered islands, such as East Ceram, Southwest Maluku, and Central Maluku. The territory of Buru and South Buru is primarily made up of land, but the island’s size, lack of road infrastructure, and limited health facilities force residents to travel by water to reach other parts of the island. Fig 4D indicates the overall health equity as a function of accessibility to health facilities and availability of health workers. Ambon is on top of the list while East Ceram is at the bottom.

## Discussion

Amid Indonesia’s rapid development, access to healthcare remains a significant challenge for some residents. In geographically challenging areas like Maluku, traveling to the nearest primary health center can be an arduous journey. Access to hospitals is another challenge, as seven out of 11 districts have only one hospital in their jurisdiction. The scarcity of health workforce in such areas has also been a major impediment to accessibility [6]. To elucidate healthcare equity among districts in Maluku Province, this study integrates the distances to puskesmas and hospitals with the number of essential health workers employed in each health facility.

Our findings reveal that Moluccans need to travel an average of 13.28 km to reach the nearest puskesmas, and 35.94 km to the nearest hospital within the district they reside. This may involve taking naval route(s) if infrastructure for land transportation is not available. The long distance is further aggravated by the absence of adequate transportation infrastructure. With the total land area of 54,185 km^2^, Maluku has only 10,433 km of road network, of which 51% are paved, 28% are dirt, and 16% are gravel. Based on their condition, 37% of these roads are classified as good, 21% are fair, 15% are damaged, and 27% are severely damaged [37]. According to the vehicle ownership statistics, the vast majority of Maluku residents use motorbikes as their main transportation [38]. In 2011, the government started to operate two bus routes that connect a few villages in Ambon, Central Maluku, and East Ceram. Today, the network has expanded into 22 routes, reaching 4 other districts (Buru, South Buru, Southeast Maluku, and West Southeast Maluku) [39].

In an archipelagic setting, water transportation facilities serve as the backbone for mobility, but are not present on all island clusters in Maluku [40]. While there are about 290 inhabited islands across the province, Maluku operates only 29 seaports and 14 vessels serving 24 inter-island routes [38]. The majority of these routes are pioneer routes, which are the services made available by the government to serve the territories not traversed by commercial vessels [38, 40]. The schedule has been reported to be relatively irregular and infrequent, with a single trip taking an average of 15 days to complete. While private boats are available for charter, the cost is generally high and unaffordable [40]. Thus, residents in isolated island clusters have to rely on smaller speedboats or fishing boats as their main transportation mode, but these vessels could not be used for long-distant routes and their operation is highly dependent on weather conditions [22].

In Indonesian health systems, individual health services are delivered based on medical needs starting from the primary care level [36]. Secondary and tertiary level services at hospitals can only be provided after referral from primary health care facilities. Given the importance of primary care in health service provision, adequacy of health workforce in puskesmas is therefore a critical issue. The Ministry of Health has regulated that the minimum standard of essential health workforce in an outpatient puskesmas should include one physician, five nurses, and four midwives, and two physicians, eight nurses, and seven midwives in puskesmas with inpatient services [11]. Using this regulation as standard, we found that nearly 77% of Puskesmas in Maluku have adequate nurses, while 65% still lack doctors and 49% lack midwives.

Furthermore, it is also noteworthy that 55% of the physicians practicing in hospitals are general practitioners, while a large number of puskesmas are in need of physicians. This situation indicates an improper distribution of human resources across health facilities. GPs are first-line physicians mainly equipped to care for common illnesses, manage ongoing health problems, and conduct preventative care. Therefore, placing too many of them in hospitals where more specialist care should take place may be less appropriate.

Primary health care is expected to manage a number of non-specialist diagnoses derived from the national competency standard for GP [41]. With the absence of physicians, combined with amenities shortages, puskesmas in Maluku could only manage 90 of 144 non-specialist diagnoses, resulting in case referrals that otherwise could actually be handled at the facility [42]. Besides incurring higher costs to the health systems, this situation also burdens patients with unnecessary travel expenses and time. This undoubtedly forces some patients to forego care altogether, when such additional travel is not feasible [10, 43]. Furthermore, it may result in an unpleasant experience that deters them from seeking care in the future [44].

Another factor that must be considered is the adverse effect of travel distance on health outcomes. Life expectancy in Maluku is five years lower than the national average (66.04 compared to 71.61) and ranks the second lowest among 34 provinces [45]. As accessibility increases, we would expect health outcomes such as life expectancy to increase. We can also infer that all-cause mortality rates should decrease as healthcare becomes more accessible.

Persistent disparity in basic public services, such as health and education, has adversely affected the quality of human resources in the peripheral areas, making them relentlessly dependent upon the supply of health workers from other regions [46]. Over the years, the Ministry of Health has launched a number of programs that send health workers to those areas, however, these efforts are often not accompanied by strategies to retain them. Low retention rates of health workforce in rural puskesmas, particularly in island regions, are attributable to lack of safety, poor living conditions, little financial incentive, and low opportunity for career development [12].

The overall healthcare equity model ranks Ambon first among other districts, followed by Tual and Southeast Maluku. As the capital of Maluku Province, Ambon is relatively more developed, heavily populated and accommodates the majority of hospitals (10 of the total 28), therefore attracting more health workers that are inclined to stay. Its small territory and good road network also make access to health facilities very easy. On the other end are East Ceram and Southwest Maluku. The two districts occupy a large area with scattered population, but only have a handful of puskesmas and one hospital in each district.

In comparison to the more traditional 2SFCA method, our results are largely similar. However, the fundamental inputs for traditional FCA require values that are well-established (e.g. distance traveled via road network, accurate population estimates per administrative division, etc.). Our study lacks several of those established metrics, and as a result, we utilized a modified and custom approach instead. That being said, the 2SFCA results do provide relatively accurate accessibility measures within each 10 km buffer, but the unknown values in white located outside those buffers were not reliable to report (S1 Fig).

Our results exemplify the importance of geospatial analysis in defining overall healthcare equity. Ambon has the lowest ratio of puskesmas health workers of all districts but shortest distance to reach health facilities, placing it first in healthcare equity. Buru and South Buru, on the other hand, have a relatively high ratio of health workers in hospitals, but the longer distance in reaching health facilities makes the overall equity poor. Similarly, East Ceram, although it appears to have a better ratio of health workers in both puskesmas and hospitals than many other districts, ranks severe in healthcare equity due to the great distances required to visit health facilities. On the contrary, the shorter overall distance rates West Ceram as having good healthcare equity despite the lack of health facilities and health workers.

These challenges remain a critical consideration in light of the Indonesian government’s efforts to achieve universal health coverage by offering free healthcare to all residents. When distance remains a significant concern, unmet needs will remain high since travel expenses and time will impede people from accessing necessary care, despite the fact that it is provided free of charge. According to the 2018 National Basic Health Research, households in Maluku Province have comparable perceptions of the difficulties of accessing puskesmas with respect to type of transportation, cost incurred, and travel time (22.92% easy, 35.66% difficult, 41.42% very difficult) and hospitals (26.77% easy, 28.56% difficult, 44.67% very difficult) [47]. Currently, 85% of Maluku’s population is eligible for free healthcare [48], but a similar proportion of population also has difficulties getting to health facilities.

The healthcare equity findings also correlate with the national public health development index issued every five years by the Ministry of Health. The index comprises a set of indicators that represent maternal and child health, communicable and non-communicable diseases, reproductive health, nutrition, as well as health determinants, such as environmental factors and risky behaviors. Ambon and Southeast Maluku consistently ranked first and second in 2013 and 2018, while Tual rose from sixth in 2013 to third in 2018. However, Ambon’s national rank had dropped from 95^th^ in 2013 to 241^st^ in 2018 (out of 514 districts), indicating that the district had not progressed as quickly as the others. East Ceram, on the other hand, continuously ranked the lowest in Maluku Province (10^th^ in 2013 and 11^th^ 2018) and was one of Indonesia’s worst-performing districts (494 from 514) [49].

We acknowledge several limitations in our study. In the spatial analysis, we only took distances, type of routes, and health human resources into consideration. However, there are many other factors that influence the ability of puskesmas and hospitals to provide appropriate services, such as catchment area, number of population served, medical equipment, and the availability of basic utilities (electricity, clean water, telephone) [10]. We were also unable to take the extreme topography of the Moluccan archipelago, absence of adequate infrastructure, and weather conditions into stronger consideration.

Nonetheless, this study demonstrates how geospatial methods provide critical tools for estimating global healthcare accessibility. GIS was integral in discovering missing residential data by overlaying high-resolution satellite imagery that was then traced, allowing for area calculations and population estimates that would not have otherwise been available. GIS also provided more reliable estimates for routes that would be taken for rural and isolated populations, by tracing dirt roads and paths that are not otherwise recorded. Lastly, GIS allowed for all this newly added spatial data to be seamlessly integrated into the same data frame, where units of measurement can be calculated and translated into visual representations (e.g. choropleth maps).

Our study serves as a novel framework for leveraging publicly available data for the practical use of geospatial analysis to monitor the ability of citizens to access healthcare in an archipelagic setting. For the disperse and rural Maluku Province especially, based on our findings we propose the following recommendations to federal and local authorities: (1) Allow and encourage cross-district referrals, in which patients can be referred to the closest hospital regardless of district administration. Eliminating the district restriction is a no-cost policy solution that would substantially reduce the time, distance, and cost for some patients to travel to hospitals. (2) Assign health workers to puskesmas that have yet to meet the standard required by the national regulation [11]. This can be done partly by redistributing the GPs in hospitals to puskesmas, as more specialists are added to the hospitals. (3) Explore the deployment of cost-effective technological solutions such as telemedicine [10], to provide interactive access to physician expertise for patients in remote areas. (4) Increase efforts to encourage healthy behaviors and lifestyles to reduce the needs for hospital visits. (5) Build hospitals or deploy floating puskesmas/hospitals to places where access to such facilities is difficult to reduce unmet needs, especially basic essential healthcare. (6) Conduct further GIS research that includes the availability of medical equipment and amenities, actual sailing routes, travel time, and topography.

Having access to the latest and most accurate maps regarding healthcare accessibility allows the government to optimally respond to emerging public health challenges and plan for improvements, including expansion of healthcare infrastructure, effective health workforce distribution, and other alternative solutions for the regions with low accessibility. Such spatially informed insights can also encourage and direct beneficial development outside of the health sector, such as adding more road network and redesigning public transportation routes and schedules. Ultimately, spatial analysis can be a powerful tool for providing policymakers at both national and local levels with evidence-based geographic information, to enable proper decision-making for resource allocation and ensure that no one in need is left behind.

## Supporting information

S1 Fig Results of puskesmas (A) and hospital (B) healthcare capacity estimated by the 2-step floating catchment area (2SFCA) method. Healthcare accessibility, usually calculated as driving distance, has been replaced with a 10 km buffer around each respective healthcare facility, as a reasonable metric for travel by walking, motor bike, and less commonly, automobile. Base map provided by GADM https://gadm.org/maps/IDN.html. License https://gadm.org/license.html.
(TIF)

S1 Table Estimates of population and number of individuals requiring land or naval routes to access healthcare facilities by district within Maluku Province. (XLSX)

S2 Table Network analysis results and raw data providing the number of routes and distances required for citizens to travel from residential areas to puskesmas by land. (XLSX)

S3 Table Network analysis results and raw data providing the number of routes and distances required for citizens to travel from residential areas to the nearest dock or port for naval travel to nearest puskesmas. (XLSX)

S4 Table Network analysis results and raw data providing the number of routes and distances required for citizens to travel from the nearest dock or port to the nearest port closest to a puskesmas (on another island). (XLSX)

S5 Table Network analysis results and raw data providing the number of routes and distances required for citizens to travel from residential areas to hospitals by land. (XLSX)

S6 Table Network analysis results and raw data providing the number of routes and distances required for citizens to travel from residential areas to the nearest dock or port for naval travel to nearest hospital. (XLSX)

S7 Table Network analysis results and raw data providing the number of routes and distances required for citizens to travel from the nearest dock or port to the nearest port closest to a hospital (on another island). (XLSX)

S8 Table Number of health care workers (physicians, nurses, and midwives) by healthcare facility (puskesmas and hospitals) by district in Maluku Province. (XLSX)

S9 Table Overall healthcare capacity (total number of physicians, nurses, and midwives) by district, hospital (within district), and puskesmas (within district). (XLSX)

S10 Table Overall distance (km), type, and condition of road networks by government administrative level. (XLSX)

S1 Text (DOCX)
