# Supplementary figures and images for: Spatial evaluation of healthcare accessibility across archipelagic communities of Maluku Province, Indonesia
Source: PLOS Glob Public Health. 2023 Mar 9;3(3):e0001600. doi: 10.1371/journal.pgph.0001600 (PMC10021735; doi:10.1371/journal.pgph.0001600)

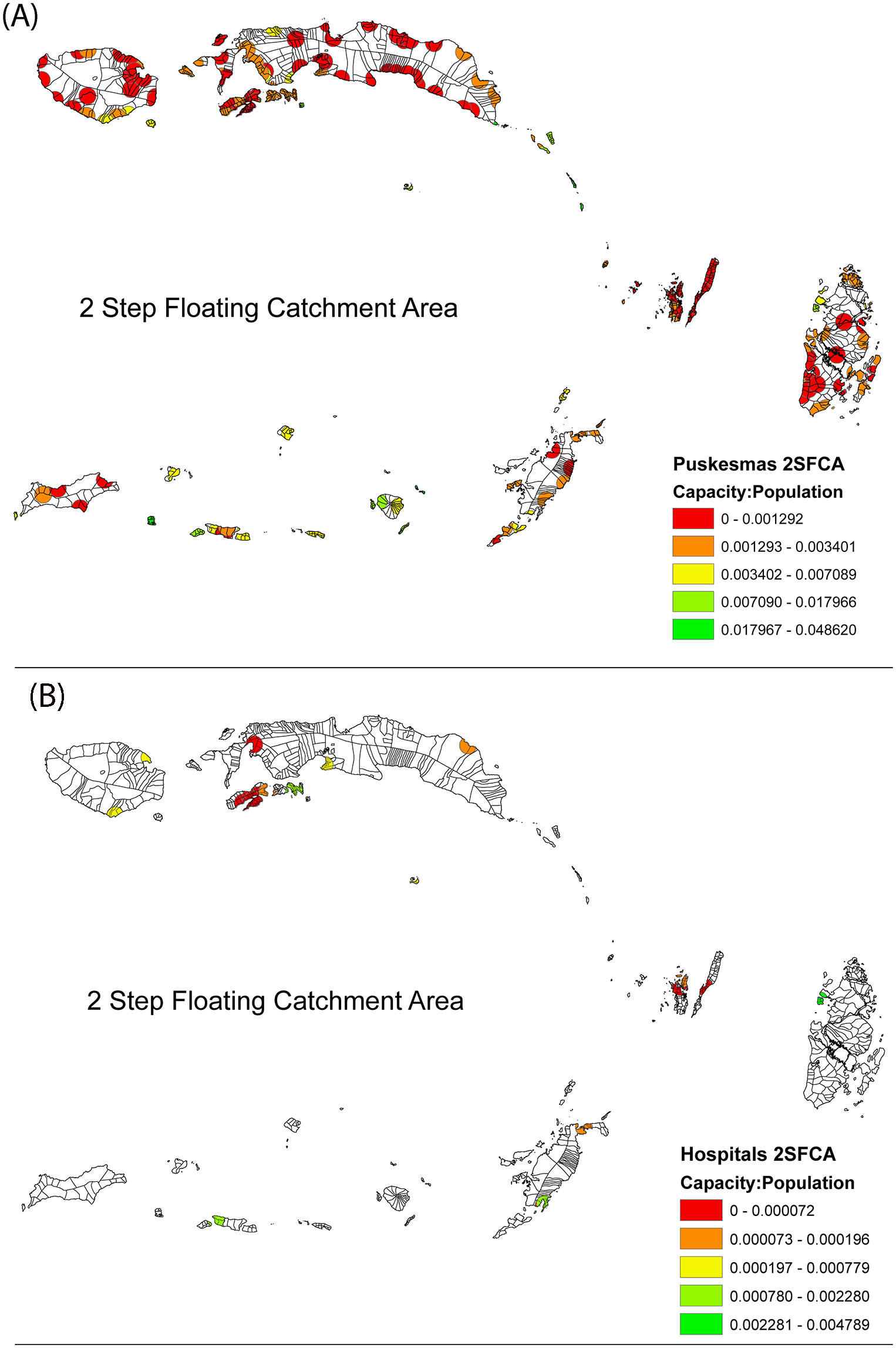

Supplement: S1 Fig — Healthcare accessibility, usually calculated as driving distance, has been replaced with a 10 km buffer around each respective healthcare facility, as a reasonable metric for travel by walking, motor bike, and less commonly, automobile. Base map provided by GADM https://gadm.org/maps/IDN.html. License https://gadm.org/license.html. (TIF) [file pgph.0001600.s001.tif]
