# Supplementary material for: Spatial evaluation of healthcare accessibility across archipelagic communities of Maluku Province, Indonesia
Source: PLOS Glob Public Health. 2023 Mar 9;3(3):e0001600. doi: 10.1371/journal.pgph.0001600 (PMC10021735; doi:10.1371/journal.pgph.0001600)
Supplement: S1 Text — (DOCX) [file pgph.0001600.s012.docx]

# Supplemental Methods

*2-Step Floating Catchment Area (2SFCA) Estimations.*

Population estimates were calculated at the sub-district level. Each sub-district’s proportion of land per district was multiplied by the total population of each district. Catchment areas were calculated as 10 km buffers around each hospital and puskesmas, respectively. 10 km was selected as a reasonable high-end distance that most citizens in Maluku province would travel for healthcare, given the means of transportation, conditions of roads, and overall rigors in traversing mountainous terrain. Each 10 km buffer was then spatially joined to each intersecting sub-district, calculating the population-weighted sums for each overlapping region. The hospital and puskesmas healthcare capacity was estimated by denoting a value of 1 for each provider (hospitals = 1 for each physician; puskesmas = 1 for each physician, nurse, and mid-wife). The capacity was then divided by the population-weighted sums from the healthcare facility’s catchment area overlay. This methodology makes two assumptions: 1. populations are evenly distributed across districts, and 2. 10 km is the maximum distance that Maluku residence would travel for healthcare.
